# Blockchain and Internet of Things Technologies for Food Traceability in Olive Oil Supply Chains

**DOI:** 10.3390/s24248189

**Published:** 2024-12-22

**Authors:** Vassilios Vitaskos, Konstantinos Demestichas, Sotirios Karetsos, Constantina Costopoulou

**Affiliations:** Informatics Laboratory, Agricultural University of Athens, 11855 Athens, Greece; stud416062@aua.gr (V.V.); karetsos@aua.gr (S.K.); tina@aua.gr (C.C.)

**Keywords:** blockchain, smart contracts, food traceability, Internet of Things (IoT), supply chain, olive oil

## Abstract

This study presents a blockchain-based traceability system designed specifically for the olive oil supply chain, addressing key challenges in transparency, quality assurance, and fraud prevention. The system integrates Internet of Things (IoT) technology with a decentralized blockchain framework to provide real-time monitoring of critical quality metrics. A practical web application, linked to the Ethereum blockchain, enables stakeholders to track each stage of the supply chain via tamper-proof records. Key functionalities include smart contracts that automate quality checks, ensuring data integrity and providing immediate verification of product authenticity. Initial user feedback highlights the system’s potential to enhance transparency and reduce fraud risks in the olive oil market, supporting consumer trust and regulatory compliance. This approach offers a scalable solution adaptable to other high-value agricultural products, demonstrating the blockchain’s transformative potential for secure and transparent food traceability.

## 1. Introduction

The agri-food industry today confronts significant challenges in maintaining food safety and quality, intensified by an increased consumer demand for transparency and traceability from farm to fork. Annually, over 23 million people in Europe suffer from food-related illnesses, leading to more than 5000 fatalities, underscoring the urgency for robust food safety solutions [1]. Blockchain technology emerges as a promising innovation in this context, offering a distributed, decentralized, and immutable database system that enhances data integrity and security without relying on a central authority [2].

This technology is gaining traction among researchers and industry stakeholders due to its potential to overcome traditional data management drawbacks, such as susceptibility to tampering and limited accessibility. Current agri-food supply chains, often minimally digitized and reliant on paper records or closed electronic systems, are ripe for enhancement. These systems are typically accessible only to specific regulatory bodies, thus limiting the overall transparency and efficiency of the supply chain [3,4]. The lack of real-time, tamper-proof data hampers efforts to verify product origin and quality, creating vulnerabilities that fraudsters can exploit. This situation underscores the need for innovative solutions that can enhance traceability and restore consumer confidence.

The integrity of high-value food products, such as extra virgin olive oil (EVOO), is particularly at risk. EVOO is prized for its nutritional properties and commands a high market price, which unfortunately also makes it a prime target for fraud. Adulteration practices, such as mixing EVOO with cheaper or inferior oils, are rampant. Recent data from the European Union highlights a troubling increase in olive oil fraud and mislabeling incidents, with 50 cases reported in the first quarter of 2024 alone, a more than threefold rise compared to the same period in 2018 [5,6]. Moreover, some estimates suggest that up to 80% of the Italian EVOO market might be affected [7]. This surge in fraudulent activities not only undermines consumer trust but also poses substantial economic threats to legitimate producers. Incidents like the arrest of individuals for exporting counterfeit EVOO in the U.S. [8] and the seizure of large quantities of adulterated oil [9] highlight the scale of this issue. The globalization of food markets further amplifies these risks, making the adoption of technologies like blockchain more crucial than ever in ensuring the authenticity and quality of food products across global supply chains [10].

Overall, blockchain’s introduction into the agri-food sector could significantly revitalize existing systems, helping to ensure that food safety regulations are met and that the quality of products like EVOO is maintained from production to consumption. Blockchain’s decentralized and immutable ledger offers unique advantages for high-value products like olive oil, where authenticity is critical. By securely recording every transaction and movement within the supply chain, blockchain can provide an unalterable history of a product’s journey from producer to consumer.

Despite the potential benefits, the application of blockchain in agricultural supply chains remains underexplored, especially concerning its integration with Internet of Things (IoT) devices for real-time data collection. This study aims to bridge this gap by developing a blockchain–IoT traceability system tailored specifically for the olive oil industry. The proposed system leverages IoT sensors to monitor critical quality metrics and employs smart contracts to automate quality verification processes, ensuring data integrity and enhancing transparency across the supply chain.

By focusing on the unique requirements of the olive oil sector, this research contributes to the broader discourse on blockchain applications in agriculture, offering insights that could be adapted to other high-value products facing similar traceability challenges.

In this light, the contribution of this paper is that it introduces a blockchain–IoT traceability system specifically designed for olive oil, addressing unique challenges in maintaining product quality and authenticity in a highly susceptible market. While blockchain and IoT applications have been widely explored in food traceability, this study presents a focused implementation on olive oil, a product frequently targeted by fraud. Key contributions of this research include:

- **Tailored Traceability Framework for Olive Oil**: The proposed system monitors olive oil at each critical stage of the supply chain using real-time IoT data inputs, including chemical composition, temperature control, and Protected Designation of Origin (PDO) verification (PDO refers to a quality label granted to products that are produced, processed, and prepared in a specific geographical area using recognized know-how). This provides transparency and quality assurance specifically suited to olive oil.
- **Smart Contract-Enabled Quality Control**: Unlike other traceability frameworks that use blockchain solely for data recording, the proposed approach integrates smart contracts to automate and enforce quality checks, such as acidity and temperature thresholds. This automation minimizes the need for manual intervention and ensures data consistency.
- **Scalable Architecture for Adaptability**: The architecture is designed with scalability in mind, allowing other high-value agricultural products to adopt a similar framework by modifying smart contracts and data inputs according to specific industry requirements.

This research thus moves beyond theoretical blockchain solutions, demonstrating a practical system tailored to olive oil’s particular traceability needs, and highlights its potential scalability for broader agricultural applications. More specifically, this study investigates critical vulnerabilities in the olive oil supply chain and evaluates the potential of a blockchain–IoT traceability system to address these challenges.

The remainder of this paper is structured as follows: Section 2 provides details about the research objectives of this study. Section 3 explores the potential of blockchain technology in enhancing traceability within the agri-food supply chain, emphasizing how the decentralized and immutable nature of blockchain secures the integrity of product information. Section 4 examines the synergy between blockchain and the Internet of Things (IoT), discussing how IoT devices provide real-time data crucial for product monitoring and addressing the challenges associated with centralized architectures, such as security vulnerabilities and performance issues. Section 5 reviews related work, offering an overview of various implementations of blockchain in supply chain management, particularly in the agri-food sector, while highlighting the unique contributions of this paper in relation to existing research. Section 6 provides a comprehensive description of the olive oil supply chain, identifying key points vulnerable to fraud and outlining how blockchain can improve transparency and security at each stage. Section 7 presents the technical implementation of the proposed blockchain-based traceability system, detailing the design of the smart contract, the server-side processes, and the client interface, all built on the Ethereum blockchain. Moreover, in this Section, the system’s effectiveness is assessed through a survey involving users in the olive oil supply chain, providing insights into its usability, operational impact, and alignment with market needs. Finally, Section 8 concludes the paper by summarizing its contributions and discussing the challenges, limitations, and potential directions for future work.

## 2. Research Objectives

This study is guided by the following research questions, which address both the vulnerabilities in the olive oil supply chain and the specific elements required to establish a blockchain-based traceability solution:

**RQ1: What are the critical points across the olive oil supply chain that are most vulnerable to fraud, and what types of smart contracts can be designed to address these vulnerabilities?** This question identifies areas in the supply chain where traceability is most needed, focusing on high-risk points susceptible to fraud. It further explores how customized smart contracts can mitigate these risks, securing the authenticity and quality of olive oil at each stage.

**RQ2: What types of data need to be tracked and recorded through IoT and blockchain technologies to ensure comprehensive traceability in the olive oil supply chain?** This question examines the essential data points that must be consistently monitored and recorded to provide transparent, real-time traceability. By integrating IoT and blockchain technologies, data collected from various stages remain immutable and accessible for validation.

**RQ3: What specific data points and smart contracts should be implemented to create a Minimum Viable Product (MVP) of a blockchain-based traceability system for olive oil?** This question identifies the core functionalities necessary to launch an MVP, focusing on the fundamental data and smart contracts required to establish a secure, efficient, and adaptable system for olive oil traceability.

**RQ4: What is the potential of a blockchain solution for olive oil traceability in terms of effectiveness, operational efficiency, transparency, scalability, and adaptability?** This question assesses the broader impact and scalability of the system, investigating how effectively it improves traceability, operational efficiency, and transparency. Additionally, it evaluates the system’s adaptability for other high-value agricultural products with similar traceability requirements.

## 3. Exploring the Potential of Blockchain Technology in Enhancing Traceability

Blockchain technology has been increasingly adopted across various high-value supply chains to address traceability and transparency challenges. In the luxury goods sector, blockchain systems are used to authenticate items like diamonds and designer handbags, ensuring that each product can be traced back to its origin and certified for authenticity [11]. For example, Everledger uses blockchain to track the provenance of diamonds, protecting both consumers and suppliers from fraud by securing each transaction with an immutable ledger. Similarly, the pharmaceutical industry has leveraged blockchain for drug traceability to prevent counterfeit medications from entering the market [12]. Companies such as IBM and Merck have developed blockchain systems to track medications from manufacturer to consumer, enhancing safety and regulatory compliance.

Blockchain technology has the potential to significantly enhance traceability in the food supply chain [13] by providing a comprehensive record of every stage of the process. It ensures transparency and reliability by securely recording vital information such as product origin, storage and transport conditions, treatments administered, and associated certifications.

For example, in the wine industry, another high-value product susceptible to fraud, blockchain solutions are used to authenticate vintages and provide consumers with verifiable information on the production process [14,15]. For instance, the company VinChain tracks each stage of the wine supply chain, allowing consumers to verify the provenance and quality of the wine they purchase.

The above examples highlight blockchain’s potential to enhance traceability and transparency across supply chains where authenticity is critical. By leveraging blockchain’s immutable ledger, these systems can securely document each step in the product lifecycle, from production to end-user, thus safeguarding against fraud. With the adoption of blockchain technology, consumers gain full visibility into and assurance of the quality and safety of the food they consume. They are able to track the origin of products, verify compliance with proper storage and transport protocols, and validate adherence to various standards and certifications, including those concerning organic agriculture.

Furthermore, blockchain technology can play a crucial role in identifying and resolving issues within the supply chain, such as epidemics or accidents. It enables rapid identification of the source and destination of products, thereby enhancing the ability to limit the spread of problems and remove unsafe batches of food from circulation.

The use of blockchain for food traceability not only enables consumers to make informed purchasing decisions but also promotes public health guarantees and instills confidence in the food supply. Additionally, traceability is essential in combating counterfeiting and fraud by ensuring that every transaction and activity within the supply chain is thoroughly documented in an anonymous yet seamless manner.

Overall, the application of blockchain technology to food traceability can markedly improve the quality, safety, and trustworthiness of the food supply chain. Consumers can have access to comprehensive information about the journey of their food, while producers, suppliers, and traders could enhance transparency and build trust in their operations. Blockchain technology can transform the entire process of food production, distribution, and consumption, thereby providing increased levels of safety, security, and trust for all parties involved [16].

## 4. Integration of loT and Blockchain

In the agricultural sector, IoT proves invaluable by aiding in the early detection of plant or animal diseases and facilitating real-time information exchanges between consumers and producers [17]. However, the capabilities provided by IoT are not without concerns. Issues such as security vulnerabilities [18], excessive data traffic, latency, and broadband challenges present significant risks [19]. These are compounded by the reliance on centralized servers for security, protection, and information processing, which can lead to service failures if the central server becomes inefficient [20].

Particularly with regards to security, IoT devices are often resource-constrained and lack robust security features, which makes them susceptible to various attacks. For instance, the Mirai botnet attack in 2016 exploited default credentials in IoT devices, leading to widespread service disruptions [21]. Such incidents underscore the necessity for stringent security measures when integrating IoT with blockchain. Common vulnerabilities include weak authentication mechanisms, insecure communication protocols, and inadequate firmware updates, all of which can be exploited to compromise the integrity of the data recorded on the blockchain [22].

Further, the reliability of data coming from IoT devices is crucial; verifying the integrity of data in a centralized architecture is problematic, as it can be altered by malicious entities. This underscores the necessity for a decentralized and distributed architecture to enhance data security and reliability.

To mitigate security risks in IoT–blockchain integration, several strategies are recommended, such as implementing robust authentication mechanisms to ensure that only authorized devices can participate in the network, reducing the risk of unauthorized data injection [23], employing encryption and secure communication protocols to protect data integrity during transmission, preventing interception and tampering [24], as well as ensuring that IoT devices receive timely firmware updates that address known vulnerabilities, enhancing overall system security [25].

One effective strategy is the use of a distributed service, allowing all network participants to confirm that data remains unmodified and accurate [26]. Blockchain technology, integrated with IoT, serves as a robust framework for data management and transaction distribution. It organizes, executes, and stores data from various IoT devices, enhancing transparency, security, and traceability without third-party involvement [27]. With its decentralized data hosting, blockchain allows data to be shared publicly across multiple servers, ensuring continuous activity and accessibility, an increasing concern with centralized databases. While the blockchain’s storage capacity is relatively modest according to modern data storage standards, the transactions it handles are typically lightweight, making it well-suited for IoT device data like temperature, pressure, and humidity, which —though minimal— are crucial for operational integrity. Hardware development platforms such as Arduino, Raspberry Pi, and ESP8266, which utilize technologies like RFID and wireless sensor networks, facilitate the necessary sensing, activation, and communication functions over the Internet [28].

Adopting a more structured approach, the interaction between IoT devices and blockchain involves several technical considerations, including:

1. **Data Transmission**: IoT devices collect data (e.g., temperature, humidity) and transmit it to the blockchain network. Due to the limited computational capabilities of many IoT devices, lightweight protocols such as MQTT (Message Queuing Telemetry Transport) are often employed to facilitate efficient data transfer [29].
2. **Data Validation and Storage**: Upon receiving data, blockchain nodes validate the information through consensus mechanisms before appending it to the ledger. Given the high volume of data generated by IoT devices, scalability becomes a concern. Solutions like off-chain storage are utilized to handle large datasets, ensuring that only critical information is stored on-chain to maintain efficiency [30].
3. **Smart Contracts**: Blockchain’s decentralized approach leads directly to the concept of smart contracts, which are self-executing contracts with the terms directly written into code. Smart contracts automate processes by executing predefined actions when certain conditions are met. In IoT–blockchain systems, smart contracts can enforce rules such as quality thresholds or trigger alerts when anomalies are detected, thereby enhancing operational efficiency and reliability [21].

In the context of agriculture [31], smart contracts can automate and enforce agreements across various stakeholders in the supply chain, ensuring transparency and reducing the need for intermediaries. They can track transactions and quality metrics, manage certifications, and enable secure, automated payments based on data from IoT devices, all while ensuring data integrity and traceability across the blockchain [32].

## 5. Related Work

Marchesi et al. [33] propose an integrated strategy for blockchain application in agri-food supply chain management. Their methodology emphasizes flexibility, enabling adaptation for different agri-food producers using generic smart contracts configured via JSON files. Biswas et al. [34] introduce a blockchain-based traceability system for wine production using MultiChain, focusing on establishing a secure, private blockchain network. Shahid et al. [35] present a comprehensive solution for agri-food supply chains on the Ethereum blockchain. The system records transactions on the blockchain and interfaces with the Inter-Platform File Storage System (IPFS), providing a secure, reliable storage solution. Their research includes simulations and security evaluations of smart tokens.

Wang et al. [36] leverage the Ethereum blockchain and smart contracts for product traceability, ensuring the authenticity of transaction participants and efficient dispute resolution by recording all events and identifying responsible parties. Malik et al. [37] describe ProductChain, a permissioned blockchain managed by entities in a generic food supply chain including government bodies. It features a three-tier architecture with shards for data reliability, scalability, and controlled access for consumers. Pincheira et al. [38] propose AgriBlockIoT, a blockchain-based system integrating IoT devices for end-to-end supply chain management, validated through a farm-to-plate model using Ethereum and Hyperledger Sawtooth.

Casino et al. [39] propose a blockchain-based model for automated traceability in the food and agriculture sector, featuring a private blockchain and a fully functional smart contract evaluated through a specific use case. Tian [40] proposes a real-time food monitoring traceability system combining HACCP, blockchain, and IoT. The system utilizes BigchainDB, merging database and blockchain benefits for transparent and secure traceability. Baralla et al. [41] focus on a blockchain platform for ensuring food provenance in a smart tourist area, integrating IoT devices for cold chain management, monitoring key data like temperature and GPS locations. Marchese and Tomarchio [42] detail a blockchain solution for managing agri-food traceability. Their system, designed for scalability and high availability, is built on a Kubernetes cluster using the Node.js Fabric SDK, tailored for compatibility with existing IT frameworks.

In comparison to the existing literature, this paper offers several unique contributions and added value to the field of blockchain-based food traceability, specifically within the olive oil supply chain. While many prior studies have explored blockchain applications in agriculture, they often focus on individual components or generalized implementations for traceability. This research distinguishes itself by proposing an end-to-end solution tailored specifically to the olive oil supply chain, which is particularly susceptible to fraud and quality degradation.

A key novel contribution of this paper is the integration of smart contracts within the blockchain framework to automate and verify quality metrics at critical stages of production. By utilizing real-time data from IoT devices, the system ensures that essential quality indicators such as acidity and pesticide residues are securely recorded and permanently linked to each batch of olive oil. This level of automation not only enhances transparency but also significantly reduces the risk of tampering or human error.

Furthermore, the paper introduces a scalable architecture that seamlessly integrates traditional supply chain management systems with decentralized blockchain technology. This architecture is designed to be adaptable for other high-value agricultural products, offering a potential blueprint for broader adoption in the agri-food industry.

Finally, this study offers a practical demonstration of the proposed system by developing a web-based application connected to the Ethereum test network. This application provides a user-friendly interface for stakeholders in the supply chain to interact with the blockchain, ensuring real-time tracking, certification, and verification of olive oil quality. The practical implementation presented in this paper bridges the gap between theoretical research and real-world application, providing a foundation for future advancements in blockchain-based food traceability systems.

## 6. Olive Oil Supply Chain and Identification of Points Susceptible to Fraud

**Figure 1:**
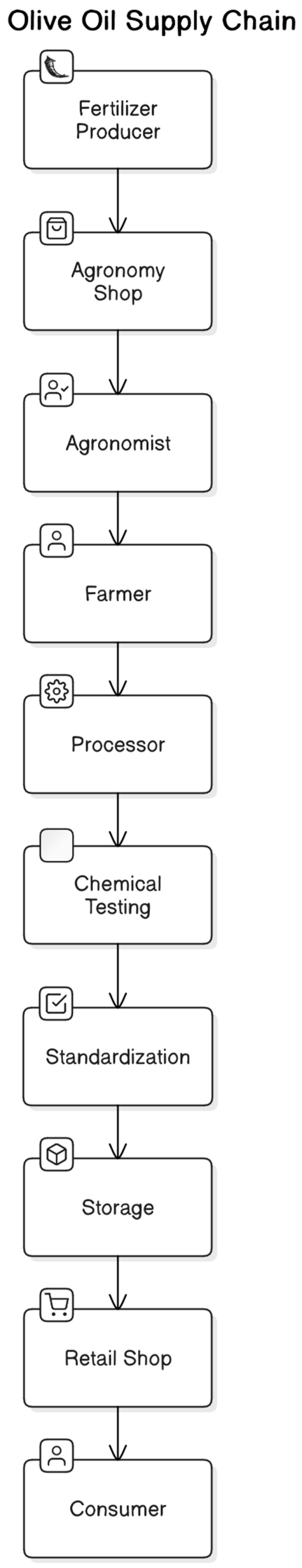
Abstract representation of the olive oil supply chain.

The olive oil supply chain involves various actors, as described in the following (Figure 1):

**(1)**  **Producer of fertilizers and insecticides:**

This actor plays a crucial role in this chain. Their responsibility is the production of fertilizers and insecticides, which will be available in agricultural shops. Packaged, the fertilizers provide detailed specifications for their safe and effective use.

These specifications, which typically include information such as application time, application method, ratio of active substances to water, and storage method, can be incorporated into smart contracts. These contracts are created to validate or invalidate the efficiency and quality of each product during the production process.

In this process, when the production is ready, the producer can compose all the smart contracts associated with each product. These contracts are initially blocked. With each sale of a product, that product moves into the pending phase, i.e., it is subject to evaluation and validation, paving the way for the continuation of the agricultural supply chain.

**(2)**  **The agronomy shop/store:**

The role of the store is focused on providing producers with full information on the usage specifications of the fertilizers and insecticides they intend to purchase. In addition, it has the obligation to ensure that the fertilizers are stored correctly, in compliance with all relevant standards.

At this point, some of the smart contracts associated with the storage of products may switch from blocked to unblocked status. For example, if storage is carried out as appropriate, a lot-based smart device can automatically validate the conditions of the storage-related smart contract. Thus, the corresponding smart contract will be moved to the unblocked status, signaling the completion of the necessary conditions for the continuation of the agricultural supply chain.

**(3)**  **The agronomist or the agricultural consultant:**

The agronomist plays a critical role in the described production chain. His/her task is to analyze farming conditions, i.e., to conduct plant and soil analyses, and to recommend the appropriate amount and type of fertilizer required by the crop of the farmer (producer) he/she is advising. At this stage, the process of purchasing the necessary formulations has been completed, including information such as the quantity of formulations, the amount of elemental macronutrients or micronutrients depending on the fertilizer, the type of formulation, and the price.

The transaction in question moves to the blockchain, enabling transparency and security. Through the blockchain, this transaction is accurately captured, ensuring the integrity and unambiguous recording of its details. The blockchain facilitates the efficient and secure management of transactions at this critical stage of the agricultural supply chain.

**(4)**  **Farmer/Producer:**

As the owner of the olive grove, the farmer takes responsibility for a multitude of agricultural processes that contribute to the production of olive oil. The farmer applies fertilizers and insecticides based on the instructions given by the agronomist and the agronomy shop, harvests the olives, and then transports the fruit to the mill. Throughout the year, the farmer fertilizes, sprays the insecticide, and carries out other necessary operations. For reasons of simplification, operations not directly related to the safety of the olive oil or for increasing its market value are not recorded.

At this point, smart contracts previously created by the manufacturer are validated in order to make verifications. For this purpose, it is convenient to connect loT devices to the fertilizer and insecticide spraying mechanisms, called chemical sensors. Chemical sensors detect and convert chemical information into electrical signals, providing qualitative or quantitative information on specific chemical components. Chemical sensors are also placed in the soil, allowing comparative analysis of the values of elements before and after fertilization.

By placing chemical sensors in the soil and performing a comparative analysis of the data, disproportionately high levels of sodium may be detected after fertilization. This may signal illegal or improper fertilizer use by the producer, and therefore the state can intervene and investigate the case based on valid monitoring information. In addition, at this point, all information regarding the date of harvesting of the fruit, place of production, and other information that increases the market value of the product is recorded. This information is provided by the Global Location Number (GLN) that identifies the physical location, legal entity, and GPS coordinates of the farmer’s estates, allowing confirmation of product origin. Specifically, if the information shows that the area is registered as a PDO, this confirms the corresponding contract created by the state PDO product certification body.

**(5)**  **The processor/elaborator:**

The processor has the role of converting the fruit into olive oil, taking great responsibility for the final quality of the product. To control the temperature, a special intelligent temperature measuring device can be used. If the temperature exceeds 27 degrees Celsius, the relevant smart contract is not validated, and this information becomes visible to the other members of the chain. These members reach decisions on the further management of the batch in question. In such cases, the product is usually not destroyed, but its value is reduced, as excessive temperature is not considered compatible with the ”cold export” label. Usually, water recycled in the softener shell is used to reduce the temperature. However, this practice can also have a negative impact on the quality of the olive oil. Consequently, each mill must carefully monitor the temperature to maintain its reliability in the production chain.

The olive oil is weighed and transferred to the storage tanks. The result of each weighing process is stored in a special block on the blockchain. If the mill has imported oil from more than one producer, each producer’s weighing is recorded separately and then incorporated into the overall result.

**(6)**  **State-certified chemical testing laboratory:**

Once the final olive oil product is produced, a trader or dealer approaches either the mill or the producer to purchase the product. To set the price, and also to put the right label on the packaging, specific analyses are carried out on the olive oil product.

The first analysis is a simple chemical analysis that gives values for acidity and K. Next, an analysis for pesticide residues is carried out. This is followed by the analysis of defects, but only if no defects are found and the chemical analysis shows values of acidity below 0.8, K268 below 0.22, and K232 below 2.5. The oil is then considered to be extra virgin olive oil. If any of these three chemical analysis indications exceed the stated limits or the median of the defects is between 0 and 2.5, then the oil is downgraded to virgin.

These processes and their results are entered into the blockchain, some manually (sensory analysis and defect analysis are performed by teams of expert tasters) and others automatically, such as chemical and pesticide residue analysis. Depending on the results, the corresponding smart contracts are validated or cancelled.

After the completion of all the above procedures, once all the contracts have been validated, the contract for the sale of the olive oil is also validated.

**(7)**  **Olive oil standardization:**

At this point, all contracts relating to the quality of olive oil have been ratified or cancelled. Depending on these contracts, the corresponding label will be affixed. The more contracts that have been validated, the higher the price of the olive oil on the shelves. If the oil is deemed unfit for consumption, it will be placed in the category of industrial oils. Before standardization, an additional check should be carried out to avoid any impurities. This check is carried out by the state-certified chemical laboratory, and automatically, once completed, it is passed to the blockchain. If this contract is not validated, the trader might face legal issues because the product does not meet the category/label that he/she wishes to apply to sell in the market.

**(8)**  **Storage/Transport:**

The conditions that transport and storage companies need to be aware of are light exposure, humidity, and temperature. All these conditions can be automatically controlled with smart devices. The data they collect are also passed to a new block on the blockchain and in turn validate three more smart contracts. Another control that could be added is regular monitoring of the condition of the product with vibration detection, data that will be shared between the transport company and the merchant.

**(9)**  **Retail shop:**

The conditions to be checked are the same as those monitored during transport and storage.

**(10)**  **Consumer:**

Finally, the consumer who chooses the product will be able to scan the barcode and see all the smart contracts’ data relating to the product, such as origin, acidity, K values, polyphenol and vitamin E content, harvest date, and any other information that can ensure the confidence of the consumer towards the product, as well as provide added value to the oil. Data such as the selling price from the producer to the trader and other sensitive data of the companies involved are not visible to the consumer.

## 7. Blockchain-Based Traceability System for Olive Oil

### 7.1. Overview of Data Required for Olive Oil Traceability

**Figure 2:**
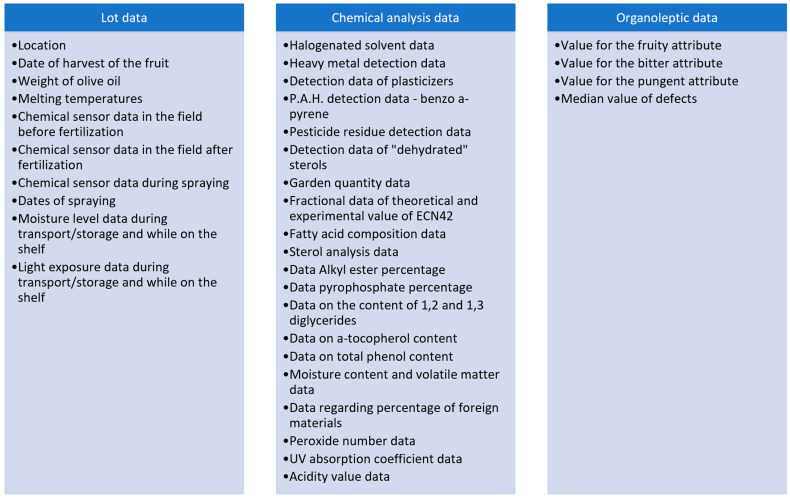
Categories and points of data for traceability in olive supply chains.

According to the description of the supply chain provided in the previous section, the data to be collected to achieve traceability in olive oil supply chains are of three main types: lot data, chemical analysis data, and organoleptic data. These are summarized in Figure 2.

All the necessary smart contracts within this supply chain can be implemented by a state actor. Before creating the corresponding smart contracts, the state actor may receive recommendations from the following blockchain entities: the fertilizer/pesticide/insecticide producer, the organic agri-food certification body, the state certified chemical laboratory, or the National Transparency Authority.

### 7.2. Technical Overview of the Proposed Blockchain-Based Traceability System

In the following, focus is placed on the part of the olive oil supply chain that features a high degree of automation, namely, the analysis of the final product before standardization; a blockchain-based traceability system tailored to the needs of that specific part is presented. The choice was made knowing that analysis laboratories have a high degree of automation, and they already store analysis results in databases. The aim has been to investigate blockchain technology on a smaller scale, with high scalability to any product tested in the laboratory and, above all, easy access to unmodifiable data. It is worth highlighting that, within a blockchain, the data are uploaded in real time and are controlled by means of smart contracts also in real time, which is important when it comes to countering fraud.

In the development of the system, two main actors were identified: a state-certified laboratory, on one hand, and end-users on the other, which may include consumers, the state, and traders who buy olive oil. The latter acquire olive oil from the producers, with a view to securing an agreement that is fully transparent and ensures fair pricing for each party.

Eleven (11) quality parameters have been defined as part of the quality assurance of olive oil, namely:

(1) Acidity;
(2) Κ268;
(3) Κ232;
(4) Median of defects;
(5) Polyphenol content;
(6) Alpha-tocopherols (vitamin E);
(7) Pesticide residues;
(8) Insecticide residues;
(9) Plasticizers;
(10) Dehydrated sterols (adulteration index); and
(11) Celsius temperature at the time of oil extraction.

**Figure 3:**
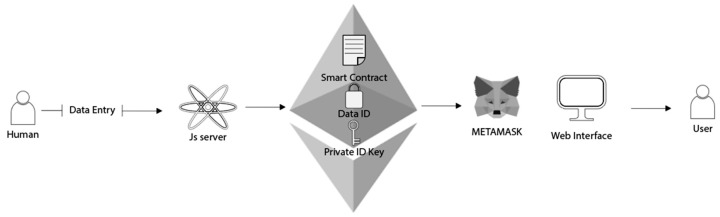
System architecture design.

The developed system aims to ensure the traceability of these quality-related attributes by creating an immutable and transparent record of quality measurements for olive oil batches. The system employs a smart contract deployed on the Ethereum blockchain, managed via a Node.js and Express software framework. The design, depicted in Figure 3, encompasses key features, including the system architecture, the server-side implementation, and the client-side interface, providing an overview of the system’s structure and functionality. Through the use of smart contracts and the server–client architecture, the system enhances traceability and ensures the confidentiality and reliability of quality assessments, addressing critical challenges in the olive oil industry.

As illustrated in Figure 3, at the core of the system is the Ethereum blockchain, interfacing through Infura and managed using a MetaMask 12.6.2 wallet for secure transaction signing. The system leverages the Ethereum blockchain due to its robust support for smart contracts, established security, and compatibility with decentralized applications (DApps). Ethereum’s smart contract functionality enables the automation of quality checks throughout the olive oil supply chain, ensuring that data integrity is maintained without relying on a central authority. While Ethereum’s transaction costs and processing speed can pose challenges, the architecture has been designed with scalability in mind, allowing for future migration to layer-2 solutions if needed to optimize performance and cost.

The blockchain component hosts the smart contract responsible for storing and managing quality data. A Node.js and Express server acts as an intermediary, facilitating communication between the blockchain and the client interface. The server processes CSV files containing quality metrics, invoking smart contract functions to record these data permanently on the blockchain. Additionally, the server provides API endpoints for retrieving and validating quality data, which are tested using Postman. The client interface, built with HTML, CSS, and JavaScript, allows users to input batch numbers and retrieve corresponding quality information. It also performs real-time quality checks by asynchronously calling the server’s API. This architecture highlights the seamless integration of blockchain technology with conventional web development frameworks, ensuring a robust, secure, and user-friendly system for quality management in the olive oil industry.

### 7.3. Smart Contract Implementation

The system’s smart contract was specifically developed to enforce quality control measures and verify product authenticity. The contract was tested through unit testing and simulation of supply chain scenarios. Unit testing verified each function individually, while simulations tested the contract’s performance under typical supply chain conditions.

In this proposed implementation, the smart contract, named **NumberChecker**, is written in Solidity and operates on the Ethereum blockchain. It manages and stores olive oil quality data using the **QualityData** struct, which encapsulates key quality parameters such as acidity, polyphenols, vitamin E, pesticides, and the other measurements, presented in Section 7.2. This structured approach ensures easy access and efficient management of quality information.

Key functions include:

- **addNumber()**: Adds or updates quality data for an olive oil batch. It accepts multiple parameters representing different quality metrics, creates a **QualityData** object, and maps it to a unique identifier (batch number). This function is important for maintaining data accuracy and traceability.
- **getNumberData()**: Retrieves quality data for a specified batch number. It includes checks to ensure that the data exists (e.g., non-zero acidity), preventing the retrieval of invalid entries.

The Solidity code is compiled using Truffle, which converts it into bytecode for the Ethereum Virtual Machine (EVM) and generates an Application Binary Interface (ABI). The ABI serves as a bridge between the blockchain and the server-side application, facilitating function calls to the smart contract. The contract is deployed to the Sepolia testnet using Truffle, HDWalletProvider, and Infura. Each time the server adds data to the smart contract, it queries the contract address, resulting in a data table similar to Table 1. HDWalletProvider handles transaction signing with private keys, securely managed through an .env file, ensuring cryptographic security and confidentiality.

### 7.4. Server-Side Implementation

The server, built with Node.js and Express, functions as an intermediary between the client interface and the blockchain. It processes CSV data, communicates with the blockchain to store or retrieve information, and serves web pages to the client.

Key server functions include:

- **processCSV()**: Reads and parses CSV files to extract olive oil quality data, converting and validating data types as needed. It invokes the smart contract’s **addNumber()** to record each batch’s data on the blockchain.

API endpoints include:

- **/getData/:number**: Handles requests to retrieve olive oil quality data for a specified batch number, by interacting with the **getNumberData()** function of the smart contract.
- **/checkQuality/:number**: Performs quality checks by retrieving data through **getNumberData()** and evaluating it against predefined quality standards.

**Figure 4:**
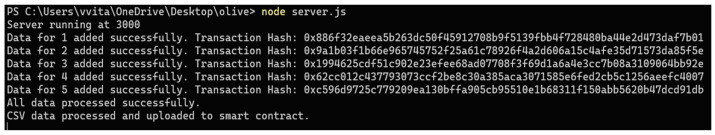
Example execution of the server-side software v0.20.1 (success message).

API endpoints are tested using Postman to simulate client requests and assess server responses. This ensures that each endpoint handles errors correctly, processes data as expected, and communicates with the blockchain without issues. The server also depicts success and failure console messages when executing, as shown in Figure 4.

### 7.5. Client-Side Interface

**Figure 5:**
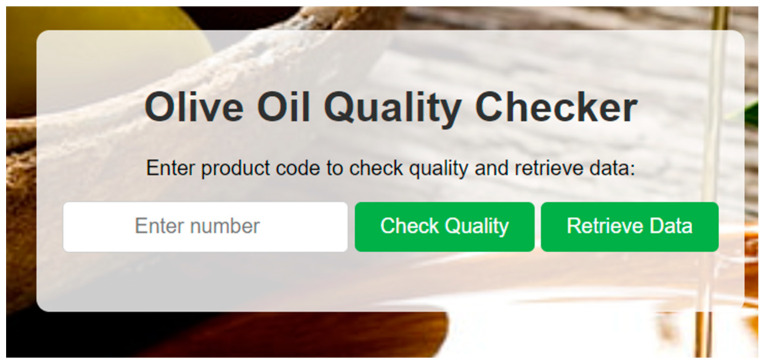
Client-side user interface of the application.

The client interface is developed using HTML, CSS, and JavaScript, providing a user-friendly environment for inputting batch numbers to retrieve information or perform quality checks, as depicted in Figure 5. JavaScript functions, such as **checkQuality()** and **retrieveData()**, are designed to make asynchronous API calls to the server, handle responses, and dynamically update the webpage to display feedback to the user.

### 7.6. User Feedback and Evaluation

To assess the initial reception and usability of the blockchain–IoT traceability system, a survey was launched and conducted, targeting key stakeholders within the olive oil supply chain. The purpose of this survey was to gather insights into how effectively the proposed system addresses user needs, enhances traceability, and contributes to consumer trust, as well as to identify potential areas for improvement. Given the critical importance of transparency and quality assurance in high-value agricultural products like olive oil, understanding user perspectives provided valuable feedback for refining the system and evaluating its market readiness.

**Methodology**: The survey was conducted over a 10-day period from 29 October to 7 November 2024 using an online questionnaire. To ensure respondents were familiar with the system, participants were shown the system in action through either a live online presentation and demonstration or a recorded video walkthrough. This approach allowed users to observe the system’s functionality, including features like smart contracts, automated quality checks, and secure data tracking through blockchain. The sample included 35 respondents, from a variety of stakeholders within the olive oil supply chain, such as producers, processors, distributors, and consumers, but primarily consisted of agronomists, who often play a central role in supply chain management and quality assurance.

**Questionnaire Structure**: The questionnaire was designed to capture user impressions of the system’s usability, impact on operational efficiency, and perceived value in enhancing traceability and consumer trust. Specifically, it comprised twelve (12) closed-ended questions and one open-ended question. Responses to the closed-ended questions were collected using a 5-point Likert scale. The scale definitions were clearly indicated and are given below for each question. This approach was chosen for its effectiveness in capturing perceptions and levels of agreement or satisfaction among participants.

**Key questions, employing a 1–5 Likert scale:**

1. How important do you consider traceability for enhancing consumer trust in the olive oil market? Likert scale definition:
  1. Not at all important
  2. Slightly important
  3. Moderately important
  4. Pretty important
  5. Very important
2. How would you rate your overall impression of using the traceability system for olive oil? Likert scale definition:
  1. Very Bad
  2. Bad
  3. Average
  4. Good
  5. Very Good
3. How easy was it to use the traceability system? Likert scale definition:
  1. Very difficult
  2. Pretty difficult
  3. Relatively easy
  4. Pretty easy
  5. Very easy
4. How easy was the process of entering and accessing data? Likert scale definition:
  1. Very difficult
  2. Pretty difficult
  3. Relatively easy
  4. Pretty easy
  5. Very easy
5. Do you believe the system positively impacts the effectiveness of traceability within the olive oil supply chain? Likert scale definition:
  1. Not at all
  2. A little
  3. Moderately
  4. Much
  5. Very much
6. Have the automated checks through smart contracts had a positive effect on reducing manual workload? Likert scale definition:
  1. Not at all
  2. A little
  3. Moderately
  4. Much
  5. Very much
7. Do you believe that blockchain technology offers greater security in monitoring the olive oil supply chain? Likert scale definition:
  1. Not at all
  2. A little
  3. Moderately
  4. Much
  5. Very much
8. To what extent do you feel the system contributes to improving olive oil quality? Likert scale definition:
  1. Not at all
  2. A little
  3. Moderately
  4. Much
  5. Very much
9. Do you believe that the traceability system adds value to the olive oil product by enhancing transparency and consumer trust? Likert scale definition:
  1. Not at all
  2. A little
  3. Moderately
  4. Much
  5. Very much
10. Do you believe that the traceability system contributes to certifying the authenticity of olive oil? Likert scale definition:
  1. Not at all
  2. A little
  3. Moderately
  4. Much
  5. Very much
11. Did you have any concerns regarding the protection of your data while using the system? Likert scale definition:
  1. Very much
  2. Much
  3. Moderately
  4. A little
  5. Not at all
12. How likely are you to use the application for future monitoring of food origin and quality beyond olive oil? Likert scale definition:
  1. Not at all
  2. A little
  3. Moderately
  4. Much
  5. Very much
13. Would you like to suggest any improvements for the future? (open-ended)

**Demographic Questions:**

- What is your role in the olive oil supply chain?
- Level of experience with traceability technologies
- Years of experience
- Age

These questions aimed to evaluate both the functional and perceived benefits of the system while also identifying any user concerns, particularly regarding data protection and usability.

**Mapping of Survey Questions to Research Questions**: Table 2 presents and explains the mapping of the survey questions to the corresponding RQs (listed in Section 2) based on their focus and relevance. In a nutshell, their relation can be summarized as follows:

- **RQ1** addresses fraud vulnerabilities and is linked to survey Questions 1, 5, 6, 7, and 10.
- **RQ2** focuses on data tracking and recording and is linked to Questions 3, 4, 5, and 8.
- **RQ3** covers MVP implementation and is linked to Questions 2, 3, 4, and 6.
- **RQ4** evaluates scalability, transparency, and adaptability and is linked to Questions 1, 2, 5, 7, 8, 9, 10, 11, and 12.

**Data Analysis**: Likert-scale responses were analyzed using descriptive statistics. Mean scores were calculated to provide an overall assessment of participant agreement with each statement. Additionally, response distributions were evaluated to identify trends or areas of divergence. It is worth noting that the computed Cronbach’s alpha (which measures how closely related a set of items are as a group, i.e., if a group of questions collectively measures the same underlying factor or construct) for questions 1–12 is approximately 0.92. This value indicates a high level of internal consistency among the questions, suggesting that they reliably measure the underlying construct (typically, a value above 0.7 is considered acceptable, and a value above 0.9 suggests excellent reliability).

**Figure 6:**
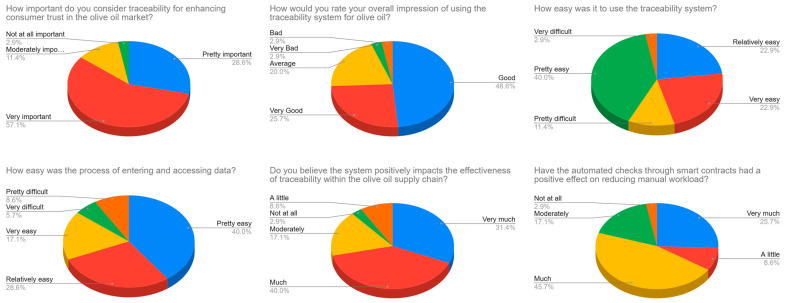

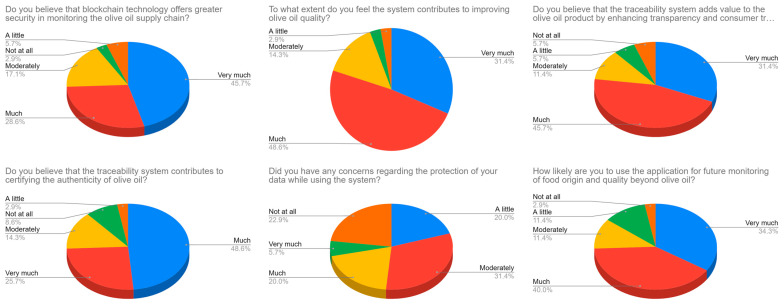
User survey response distributions (N = 35).

**Key Survey Results and Findings:** Detailed results for each survey question, including mean scores and response distributions, are presented in Table 3 and Figure 6, and discussed in the following paragraphs. The survey findings provide a detailed overview of user feedback, highlighting the system’s strengths in enhancing traceability and its alignment with market needs, as well as identifying potential areas for improvement. Key insights include a strong user perception of the system’s importance for consumer trust, high ratings for usability, and positive feedback on the impact of smart contracts in reducing manual tasks.

**Survey Results Summary**: Table 3 presents the descriptive statistics for the survey questions designed to evaluate the blockchain–IoT traceability system for olive oil. Each question measures respondents’ perceptions of various aspects of the system, with mean scores and standard deviations providing insights into overall trends and variability.

1. **High Importance of Traceability**: Question 1 received the highest mean score (4.37), indicating a strong consensus among respondents on the critical role of traceability in enhancing consumer trust in the olive oil market.
2. **Positive Impression of the System**: While the overall impression of the system (Question 2) received a moderately high mean score (3.91), responses showed slightly greater variability (SD = 0.92), suggesting room for improvement to elevate user satisfaction further.
3. **Ease of Use and Data Entry**: Questions 3 and 4, which addressed usability and the ease of data entry/access, received relatively lower scores (3.69 and 3.54, respectively). These results suggest that while the system is usable, some respondents found it challenging, highlighting a need for interface enhancements or user training.
4. **Effectiveness and Impact**: Question 5 (impact on traceability effectiveness) and Question 6 (effect of automated checks via smart contracts) both scored around 3.8, suggesting moderate satisfaction with the system’s functional benefits. These areas can be further refined to enhance operational efficiency.
5. **Blockchain Security and Quality Improvement**: Questions 7 and 8 scored above 4.0, demonstrating strong confidence in blockchain’s security and its contribution to improving olive oil quality. These results validate the system’s core strengths and its alignment with stakeholder priorities.
6. **Value Addition and Authenticity Certification**: Questions 9 and 10 focused on the added value of transparency and authenticity certification, with mean scores around 3.9 and 3.8, respectively. While generally positive, these areas may benefit from additional features to enhance perceived value.
7. **Concerns About Data Protection**: Question 11, regarding data protection concerns, received the lowest mean score (3.34) and the highest standard deviation (1.15). This indicates a wider range of opinions and underscores the importance of addressing data privacy to alleviate user concerns.
8. **Future Adoption**: Question 12 showed moderate enthusiasm for future use of the system beyond olive oil, with a mean score of 3.91. This suggests scalability and adaptability are perceived as strengths but could be further reinforced through user engagement and system enhancements.

**Detailed Survey Findings**: The full survey findings are detailed below and are derived for each survey question from the response distributions presented in Figure 6.

1. **Importance of Traceability for Consumer Trust**

**Response Summary**: 57.1% of respondents rated traceability as “Very Important”, and 28.6% as “Pretty Important”, totalling 85.7% who consider it crucial for building consumer trust.

**Insight**: This overwhelming support for traceability highlights its importance as a critical feature in high-value products like olive oil. The findings suggest that the blockchain–IoT system is well-aligned with market demands for transparency, which is essential to fostering consumer confidence and trust.

2. **Overall Impression of the Traceability System**

**Response Summary**: 48.6% rated their impression as “Good”, and 25.7% as “Very Good”, with a combined 74.3% expressing positive satisfaction. However, 20% rated it “Average”, and a few rated it lower.

**Insight**: The overall user satisfaction is high, but the system could still be optimized to convert more “Average” users to “Good” or “Very Good” ratings. This suggests potential for targeted improvements, such as enhancing usability and providing additional user support, to elevate user satisfaction.

3. **Ease of Use of the System**

**Response Summary**: 85.7% of respondents found the system “Relatively Easy” or better, with 40.0% rating it “Pretty Easy”.

**Insight**: While most users found the system accessible, the 14.3% who found it challenging point to a need for potential user interface adjustments or increased support resources. These changes could make the system even more intuitive, ensuring accessibility across various levels of technical familiarity.

4. **Ease of Data Entry and Access**

**Response Summary**: 85.7% rated data entry and access as at least “Relatively Easy”, though 14.3% found it “Pretty Difficult” or “Very Difficult”.

**Insight**: Although data entry and retrieval were manageable for most, feedback from those who struggled indicates a need for simplifying these processes further. Making data entry more intuitive could help ensure consistent data quality and enhance the overall user experience.

5. **Impact on Effectiveness of Traceability**

**Response Summary**: 74.3% indicated the system had a positive impact on the effectiveness of traceability, with 34.3% rating it “Very Much”.

**Insight**: This positive rating reinforces the system’s role in enhancing traceability, one of its core objectives. With only 5.7% seeing no positive impact, these results indicate that the blockchain–IoT system was well-received as a reliable traceability solution.

6. **Impact of Automated Checks via Smart Contracts**

**Response Summary**: 71.4% agreed that automated checks via smart contracts reduced manual workload, while 28.6% did not observe a significant impact.

**Insight**: The majority view of automation as beneficial for reducing manual tasks confirms that smart contracts add operational efficiency. The minority who reported no benefit may benefit from additional training to understand and leverage these features fully.

7. **Security of Blockchain Technology**

**Response Summary**: 85.7% agreed that blockchain technology enhances security, with 48.6% responding “Very Much”.

**Insight**: Strong support for blockchain’s security benefits indicates that users trust its effectiveness in securing supply chain information. This highlights blockchain’s role as a critical component in traceability solutions for protecting sensitive data.

8. **Contribution to Olive Oil Quality**

**Response Summary**: 62.9% felt the system contributed “Very Much” or “Much” to quality improvement, while 11.4% felt the effect was minimal.

**Insight**: Many respondents perceive an indirect quality benefit, likely due to improved data integrity and standards enforcement throughout the supply chain. This suggests that traceability contributes to maintaining product quality.

9. **Contribution to Product Value (Transparency and Trust)**

**Response Summary**: 65.7% indicated that the system added value by enhancing transparency and trust, with 31.4% rating this as “Very Much”.

**Insight**: Users see the transparency features as essential for reinforcing consumer trust and product value. This aligns with market expectations, highlighting how traceability can strengthen a brand’s reputation and appeal.

10. **Contribution to Authenticity Certification**

**Response Summary**: 71.4% believed the system supports authenticity certification, with 37.1% responding “Very Much”.

**Insight**: This high percentage reflects a positive view of the technology’s capability to certify product authenticity, further underscoring its value in combating product fraud and validating quality.

11. **Concerns about Data Protection**

**Response Summary**: 31.4% expressed moderate concern about data protection, while 22.9% had no concerns.

**Insight**: Data protection remains an important consideration, as some users have concerns about how their information is handled. Additional educational materials explaining blockchain’s security features could help alleviate these worries.

12. **Likelihood of Future Use for Monitoring Food Quality**

**Response Summary**: 74.3% of respondents are likely to use the system for monitoring food quality beyond olive oil, with 40.0% responding “Much”.

**Insight**: The high likelihood of future use indicates that users recognize the system’s potential for scalability across other products. This feedback shows that the system holds promise as a broadly applicable tool for food quality assurance.

13. **Suggestions for Improvement**

**Summary of Suggestions**: Specific suggestions included developing a platform to connect stakeholders and providing training on blockchain and IoT for small producers.

**Insight**: These suggestions emphasize the value of stakeholder connectivity and training. Addressing these areas could enhance the user experience, especially for smaller producers who may benefit from additional support in adopting the technology.

**Survey Limitations**: While the survey provided valuable insights into the system’s usability and perceived benefits, some limitations should be noted. With 35 responses primarily from agronomists, the sample may not fully represent all stakeholder perspectives in the olive oil supply chain, particularly those of retailers or distributors. Additionally, since the survey was conducted after brief demonstrations rather than extended use, respondents’ impressions may evolve over time as they become more familiar with the system. The reliance on self-reported data introduces potential biases, including social desirability or varying levels of technology familiarity, which could affect responses on usability and security. Moreover, although the high value of Cronbach’s alpha is positive with respect to the survey’s reliability, it might also be an indication of some redundancy among the questions (i.e., some questions might be asking similar things). Furthermore, the survey’s geographic reach and limited question scope may restrict the generalizability of the findings. Addressing these limitations in future studies could provide a more comprehensive evaluation of the system’s impact and effectiveness across diverse user groups.

### 7.7. Future Extensions

**Figure 7:**
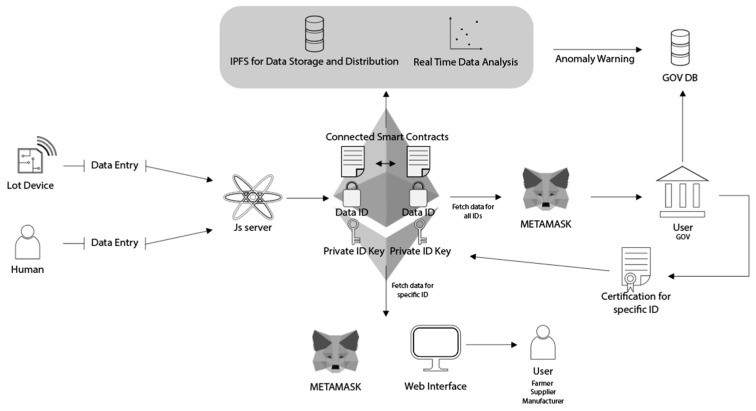
Extended and scalable architecture for a food quality and safety certification system.

The proposed system could be extended in several aspects, as illustrated in Figure 7:

**Interconnected Smart Contracts**: Developing and integrating multiple smart contracts that communicate with each other, with each managing a different aspect of the supply chain, such as production, processing, transport, and final distribution. These interconnected contracts would share a common product ID, ensuring seamless data flow across all stages.

**Digital Certificates**: Creating digital certificates for each stage of the supply chain, registered on the blockchain, to ensure the integrity and continuity of information. These certificates could be issued for specific batch IDs or for an entire product, ensuring transparency and quality assurance throughout the process.

**Real-time Data Analysis**: Integrating real-time data analysis tools capable of processing large volumes of supply chain data, providing early insights and warnings about potential quality or safety issues.

**Use of Decentralized Infrastructure Services**: Leverage decentralized hosting services, such as InterPlanetary File System (IPFS), for data storage and distribution, enhancing the security and availability of supply chain information.

**User-friendly Platform**: Developing a user-friendly platform that enables supply chain stakeholders to easily post, monitor, and verify quality data. The platform can provide support for administrative operations, in order to call specific *add* and *view* functions within the smart contracts, simplifying interaction with the system.

## 8. Conclusions

The integration of blockchain technology into the agri-food supply chain, particularly for olive oil traceability, presents a revolutionary approach to addressing long-standing challenges in food safety, quality assurance, and transparency. By leveraging blockchain’s decentralized, immutable ledger and its capacity to work seamlessly with IoT devices, this study demonstrated how food traceability can be drastically improved. Blockchain ensures that every stage of the supply chain, from production to final delivery, is securely recorded, eliminating opportunities for tampering or fraud.

To demonstrate this potential, this paper presented a practical web application linked to the Ethereum blockchain to monitor olive oil quality using smart contracts. These contracts enhance traceability by recording critical quality metrics, ensuring data integrity, and providing real-time updates across the supply chain. The decentralized nature of the system means that there is no reliance on a central authority, which is prone to performance issues and vulnerabilities, such as server outages and data breaches. In a nutshell, key benefits of this approach include:

- **Transparency and Trust**: Blockchain allows all participants, including producers, retailers, and consumers, to access real-time, tamper-proof records of the product’s journey through the supply chain. This transparency fosters trust and confidence [43], especially for high-value products like olive oil, which are frequently targeted by fraud.
- **Enhanced Traceability**: The smart contracts deployed within the system ensure that each batch of olive oil is traceable throughout its lifecycle. This feature is critical in addressing quality concerns and ensuring compliance with safety regulations, offering a more robust mechanism than traditional methods.
- **Automation and Efficiency**: Through smart contracts, the system automates key processes such as quality verification and certification, drastically reducing human error and the need for intermediaries. This reduces operational costs while speeding up decision-making.

In addition to technical findings, a survey was conducted among stakeholders to gather insights into the system’s usability, effectiveness, and overall reception. The results indicate strong support for the system’s contribution to traceability and consumer trust, with 85.7% of respondents rating traceability as “Pretty Important” or “Very Important” for enhancing consumer confidence. Overall, 74.3% of participants had a positive impression of the system, although feedback also highlighted areas for potential improvement, such as data entry and accessibility.

Considering the research questions that have been defined in Section 2, and based on the elaborate discussions of Section 7, the following conclusions can be drawn:

**Response to RQ1: Supply Chain Vulnerabilities and Smart Contract Solutions**

The study identified 10 distinct stages within the olive oil supply chain, each with its own characteristics and potential vulnerabilities. Critical points where fraud risks are highest include verifying product origin, validating storage conditions, and checking chemical quality. Survey responses emphasized the importance of traceability for consumer trust, with a mean score of 4.37 out of 5 (SD = 0.91) for its perceived importance. Smart contracts were tailored for these stages to safeguard product authenticity, such as enforcing PDO status, monitoring temperature during processing, and validating controlled storage environments. These targeted interventions directly address key fraud vulnerabilities, enabling higher transparency and quality control. By implementing these smart contracts, the system is expected to reduce fraud incidents and improve regulatory compliance across the supply chain.

**Response to RQ2: Essential Data Points for Traceability**

Comprehensive traceability requires consistent tracking and recording of specific data points, encompassing lot data, chemical composition (e.g., acidity and polyphenol levels), and organoleptic data. In total, the study identified 34 key data points (see Figure 2), which cover critical quality and traceability parameters. Questions related to ease of data entry and access received moderate scores (Mean = 3.54, SD = 1.07), highlighting room for improvement in usability. By integrating IoT sensors with blockchain’s immutable ledger, the system provides a transparent record of olive oil’s journey from production to consumption, strengthening trust among consumers and regulators. This strengthens trust and confidence among market actors, including consumers and regulators, through consistent and accessible data.

**Response to RQ3: Minimum Viable Product Requirements**

For a Minimum Viable Product (MVP), critical data points include at least 11 essential parameters to ensure olive oil traceability and quality. These include measurements of acidity, Κ268 and Κ232 absorption values, median of defects, polyphenol content, alpha-tocopherol (vitamin E) levels, and residues of pesticides, insecticides, and plasticizers. Additional indicators, such as dehydrated sterols (as an adulteration index) and extraction temperature in Celsius, further enhance the system’s capacity to verify product authenticity and processing standards. Survey participants rated their overall impression of the system at 3.91 (SD = 0.92), and automated checks via smart contracts were positively evaluated (Mean = 3.83, SD = 1.03). These findings support the MVP’s functionality while identifying opportunities for iterative improvements.

**Response to RQ4: Broader Potential of the Blockchain-Based Solution**

The system demonstrates substantial potential to enhance traceability, operational efficiency, and transparency across the olive oil supply chain. Survey respondents expressed strong confidence in the blockchain’s security capabilities (Mean = 4.09, SD = 0.88) and its contribution to quality improvement (Mean = 4.03, SD = 0.99). Additionally, 74% of respondents indicated they would use the system for monitoring other high-value food products, highlighting its scalability and adaptability. Automated checks via smart contracts, which 71.4% of respondents found useful for reducing manual workload, further support operational efficiency. The modular and scalable design of the framework reinforces its broader applicability in food traceability where authenticity and quality are critical.

In summary, the blockchain–IoT system helps to face longstanding challenges in olive oil traceability, offering a secure, scalable, and transparent solution that meets the traceability demands of high-value products. This approach not only addresses existing challenges but also anticipates the evolving demands for transparency and traceability in global agricultural markets, laying a solid foundation for future development and application across diverse sectors in agriculture where traceability and quality assurance are essential.

The implications of this research extend beyond olive oil, providing a blueprint for other industries facing similar issues of fraud, authenticity, and supply chain complexity. High-value agricultural products such as wine, strawberries, dairy, and honey could benefit from similar blockchain–IoT systems, leveraging automated quality checks and tamper-proof records to safeguard against adulteration and improve regulatory compliance.

From a policy perspective, this technology aligns well with the increasing emphasis on transparency in food supply chains. Policymakers might consider integrating blockchain-based traceability standards into regulatory frameworks, incentivizing adoption through grants or subsidies, particularly for small and medium-sized enterprises that might face cost-related barriers. Standardized traceability protocols could help harmonize data collection and reporting, strengthening consumer confidence and enabling more effective responses to food safety concerns.

In summary, the blockchain–IoT system helps to address longstanding challenges in olive oil traceability, offering a secure, scalable, and transparent solution that meets the traceability demands of high-value products. This approach not only resolves existing issues but also anticipates evolving needs for transparency in global agricultural markets, providing a strong foundation for broader application across sectors where traceability and quality assurance are crucial.

**Future extensions** of this work may involve integrating advanced real-time data analysis tools and decentralized storage solutions such as IPFS, and expanding coverage to additional supply chain stages. More interconnected smart contracts could allow for a holistic view of the supply chain, further enhancing traceability and quality assurance. Improved interconnection among stakeholders and targeted training initiatives could also support broader adoption.

Implementing these features also presents technical and cost-related challenges. From a technical perspective, managing the high volume of data generated by IoT devices remains a key consideration. To address this, solutions such as off-chain storage and layer-2 scaling (e.g., Polygon) could be explored to reduce blockchain transaction costs and improve system performance. Additionally, ensuring the security of data across multiple IoT nodes may require more advanced security protocols, particularly as the system scales to larger, more complex supply chains.

Cost-related barriers are also anticipated, particularly concerning blockchain transaction fees, IoT device maintenance, and potential infrastructure upgrades required for scalability. These expenses may impact the system’s feasibility for smaller producers, necessitating strategies to optimize costs or explore alternative funding models. Overcoming these challenges will be essential to ensure that the system is both practical and sustainable in the long term.

Regarding evaluation, building on the initial survey findings, future efforts could include a longitudinal study to observe user interactions over time, capturing any evolving perceptions and identifying long-term usability challenges. Expanding the sample to a wider range of stakeholders and regions would provide a more comprehensive view of the system’s effectiveness across the olive oil supply chain. Mixed-methods approaches, combining quantitative surveys with qualitative interviews, could yield deeper insights into user experiences and specific areas for improvement. Additionally, testing the system with other high-value agricultural products would validate its adaptability and scalability across diverse supply chains.

While challenges remain, the blockchain and IoT-powered approach to food traceability offers transformative potential for the agri-food industry. With proper investment and development, this technology can help ensure high-quality, safe products for consumers, while increasing consumer trust and quality assurance for producers. The system outlined in this study provides a foundation for innovation and development towards secure, transparent management of food supply chains.

## Figures and Tables

**Table 1:** Etherscan search data for our smart contract address (example execution).

Hash	Method	Block	Age	From	To	Value	Txn Fee
0x1745c0292d...	0xdb19338c	5917762	2 min ago	0x1390528f...92Dd8c1E3	0x865dd686...527032036	0 ETH	0.00074859
0xe034f9c4f99...	0xdb19338c	5917761	2 min ago	0x1390528f...92Dd8c1E3	0x865dd686...527032036	0 ETH	0.00077232
0xb108196428...	0xdb19338c	5917760	3 min ago	0x1390528f...92Dd8c1E3	0x865dd686...527032036	0 ETH	0.00072075
0x72c7bdf3078...	0xdb19338c	5917759	3 min ago	0x1390528f...92Dd8c1E3	0x865dd686...527032036	0 ETH	0.00069147

**Table 2:** Mapping of the study’s survey questions to the research questions.

Survey Question Number	Relevant Research Questions (RQs)	Justification
1	RQ1, RQ4	Highlights the importance of transparency and trust, aligning with the need to address fraud vulnerabilities (RQ1) and assessing broader system impact (RQ4).
2	RQ3, RQ4	Evaluates the overall usability and effectiveness of the MVP (RQ3) and its scalability/adaptability for broader application (RQ4).
3	RQ2, RQ3	Assesses usability related to system implementation (RQ3) and the data flow captured by IoT for traceability (RQ2).
4	RQ2, RQ3	Evaluates the practical implementation of data tracking and accessibility (RQ2) and its role in creating a functional MVP (RQ3).
5	RQ1, RQ2, RQ4	Explores how the system addresses vulnerabilities (RQ1), data tracking and recording (RQ2), and overall effectiveness and scalability of the solution (RQ4).
6	RQ1, RQ3	Directly evaluates the role of smart contracts in addressing supply chain vulnerabilities (RQ1) and their integration in the MVP (RQ3).
7	RQ1, RQ4	Highlights the contribution of blockchain in enhancing security (RQ1) and overall system transparency and adaptability (RQ4).
8	RQ2, RQ4	Explores how the system’s traceability ensures product quality through data monitoring (RQ2) and assesses its broader benefits and scalability (RQ4).
9	RQ4	Focuses on the added value of transparency and consumer trust, which are core components of scalability and adaptability (RQ4).
10	RQ1, RQ4	Addresses how the system ensures authenticity through data validation (RQ1) and its implications for scalability and trust (RQ4).
11	RQ4	Evaluates user concerns about data protection, a critical aspect of scalability and adaptability for broader application (RQ4).
12	RQ4	Directly explores the system’s scalability and adaptability for broader use beyond olive oil (RQ4).

**Table 3:** Descriptive statistics for the survey questions.

Survey Question Number	Question Title	Mean Score	Standard Deviation
1	How important do you consider traceability for enhancing consumer trust in the olive oil market?	4.37	0.91
2	How would you rate your overall impression of using the traceability system for olive oil?	3.91	0.92
3	How easy was it to use the traceability system?	3.69	1.05
4	How easy was the process of entering and accessing data?	3.54	1.07
5	Do you believe the system positively impacts the effectiveness of traceability within the olive oil supply chain?	3.89	1.05
6	Have the automated checks through smart contracts had a positive effect on reducing manual workload?	3.83	1.03
7	Do you believe that blockchain technology offers greater security in monitoring the olive oil supply chain?	4.09	0.88
8	To what extent do you feel the system contributes to improving olive oil quality?	4.03	0.99
9	Do you believe that the traceability system adds value to the olive oil product by enhancing transparency and consumer trust?	3.91	0.97
10	Do you believe that the traceability system contributes to certifying the authenticity of olive oil?	3.80	1.07
11	Did you have any concerns regarding the protection of your data while using the system?	3.34	1.15
12	How likely are you to use the application for future monitoring of food origin and quality beyond olive oil?	3.91	0.97

## Data Availability

Dataset available on request from the authors.
